# Activity and Safety of Bevacizumab Plus Fotemustine for Recurrent Malignant Gliomas

**DOI:** 10.1155/2014/351252

**Published:** 2014-05-04

**Authors:** V. Vaccaro, A. Fabi, A. Vidiri, D. Giannarelli, G. Metro, S. Telera, S. Vari, F. Piludu, M. A. Carosi, V. Villani, F. Cognetti, A. Pompili, L. Marucci, C. M. Carapella, A. Pace

**Affiliations:** ^1^Department of Medical Oncology, Regina Elena National Cancer Institute, Via Elio Chianesi 53, 00144 Rome, Italy; ^2^Diagnostic Oncology Department, Regina Elena National Cancer Institute, Via Elio Chianesi 53, 00144 Rome, Italy; ^3^Biostatistic Unit, Regina Elena National Cancer Institute, Via Elio Chianesi 53, 00144 Rome, Italy; ^4^Division of Medical Oncology, Santa Maria della Misericordia Hospital, Azienda Ospedaliera di Perugia, Perugia, Italy; ^5^Division of Neurosurgery, Regina Elena National Cancer Institute, Via Elio Chianesi 53, 00144 Rome, Italy; ^6^Department of Pathology, Regina Elena National Cancer Institute, Via Elio Chianesi 53, 00144 Rome, Italy; ^7^Division of Neurology, Regina Elena National Cancer Institute, Via Elio Chianesi 53, 00144 Rome, Italy; ^8^Department of Radiation Oncology, Regina Elena National Cancer Institute, Via Elio Chianesi 53, 00144 Rome, Italy

## Abstract

*Background*. No established chemotherapeutic regimen exists for the treatment of recurrent malignant gliomas (rMGs). Herein, we report the activity and safety results of the bevacizumab (B) plus fotemustine (FTM) combination for the treatment of rMGs. *Patients and Methods*. An induction phase consisted of B 10 mg/kg days 1, 15 plus FTM 65 mg/m^2^ days 1, 8, and 15. Nonprogressive patients entered the maintenance phase with B 10 mg/kg plus FTM 75 mg/m^2^ every 3 weeks. The primary endpoint was response rate; secondary endpoints included safety, progression free survival (PFS), and overall survival (OS). *Results*. Twenty-six patients affected by recurrent MGs (50% glioblastoma) were enrolled. Eight partial responses (31%) were observed. Median PFS and OS were 4 (95% C.I.: 2.8–5.1) and 6 months (95% C.I.: 4.2–7.8), respectively. Responses were significantly associated with both improved PFS and OS (*P* = 0.002 and *P* = 0.001, resp.). Treatment adverse events were mostly mild to moderate in intensity. Bevacizumab-related adverse events included grade 3 venous thromboembolic event (8%), grade 2 epistaxis (4%), hypertension (8%), and gastrointestinal perforation (4%). *Conclusions*. Bevacizumab plus FTM showed activity and good tolerability in pretreated MGs. Further investigations are needed in order to verify the benefits deriving from the addition of B to a cytotoxic in this clinical setting of patients.

## 1. Introduction

Malignant gliomas (MGs) account for roughly 50% of all malignant primary brain tumors in adults. Active treatments include resection of the tumor to the extent that is surgically feasible, radiotherapy, and chemotherapy. Glioblastoma multiforme (GBM) represents the most common form of malignant gliomas [1]. Its aggressive clinical behavior leads to a dismal prognosis, with a median survival of approximately 14 months. The standard of care for surgically resected patients with newly diagnosed GBM is based on the concurrent administration of radiation plus temozolomide (TMZ) followed by TMZ maintenance up to 6 months [2, 3]. However, long-term survival is rarely achieved and the disease almost invariably recurs. Nowadays, there are no established therapeutic options for the treatment of rMGs. Nitrosourea compounds such as carmustine, Lomustine, and the third generation nitrosourea derivative fotemustine (FTM) are associated with response rates ranging from 15 to 30% and a median progression free survival (PFS) that rarely exceeds 6 months [4, 5]. Notwithstanding, FTM has been shown to cross the blood-brain barrier because of its lipophilic profile and in the population of patients with recurrent GBM demonstrated a good activity and a favorable safety profile, mielosuppression being the most prevalent side effect [4–8].

Recent progress in the understanding of the molecular characteristics of GBM has prompted the development of targeted therapeutic approaches. As compared to normal brain tissue, rMGs show an intense vascular proliferation with high expression of the vascular endothelial growth factor (VEGF) [9]. Vessel density degree and VEGF level expression have been shown to directly correlate with the biologic aggressiveness of gliomas and a worse prognosis [10–13]. Furthermore, it has been demonstrated that VEGF inhibition leads to a decreased growth of glioma cell lines and that antiangiogenic agents can reduce both peritumoral edema and the need for corticosteroid therapy [13, 14]. On this basis, a strong rationale existed for the use of bevacizumab, an anti-VEGF monoclonal antibody, for treatment of rMGs (recurrent malignant gliomas). A bevacizumab combination regimen was first evaluated in recurrent GBM in association with irinotecan in either prospective phase II or retrospective studies that showed encouraging response rates and relevant clinical benefit (CB) [9, 11, 12, 15, 16]. Also, the activity of bevacizumab has been demonstrated when administered as a single agent [12].

Currently, few data are available on the combination of bevacizumab with nitrosoureas; therefore, we conducted an observational prospective study evaluating the activity and safety of bevacizumab plus FTM for the treatment of recurrent MG patients.

## 2. Materials and Methods

Patients with histologically proven MGs and clinicoradiological progression after no more than two previous chemotherapy lines, temozolomide plus radiotherapy, must have been the upfront therapy. Progression had to be documented by magnetic resonance imaging (MRI). Inclusion criteria were evaluable and/or measurable disease; at least 12 weeks from a second intracranial surgery and/or radiotherapy and 4 weeks from first or second-line chemotherapy; age between 18 and 80 years; Karnofsky performance status (KPS) ≥ 60; and adequate hematological, liver, and renal function. Treatment with low dose of heparin for antithrombotic prophylaxis was permitted. Previous treatment including either bevacizumab or FTM and evidence of hemorrhage at baseline MRI excluded patients from the study. Other exclusion criteria were pregnancy or nursing, clinically significant cardiovascular diseases, such as congestive heart failure (NYHA classes II, III, and IV), unstable angina pectoris or myocardial infarction within 6 months prior to study entry, uncontrolled hypertension (systolic blood pressure > 150 mmHg and/or diastolic blood pressure > 100 mmHg on treatment) or history of hypertensive crises or hypertensive encephalopathy, history of stroke or transient ischemic attack within 6 months prior to study entry, clinically significant vascular disease or symptomatic peripheral vascular disease, presence or history of recurrent thromboembolism (>1 episode of deep venous thrombosis or peripheral embolism) during the past 2 years, inherited bleeding diathesis or coagulation disorder, intestinal perforation or the presence of other condition that would have made the treatment unsafe, active infections or other uncontrolled diseases, and psychiatric disorders. The trial was conducted in agreement with the Declaration of Helsinki and International Committee on Harmonization guidelines for good clinical practice. All enrolled patients were amenable to compliance with testing and were informed of the investigational nature of the study. The study was approved by a local ethics committee and a signed informed consent was obtained by the patients.

### 2.1. Treatment Plan

All patients received FTM in combination with bevacizumab. The treatment consisted of an induction phase with bevacizumab i.v. at the dose of 10 mg/kg every three weeks plus FTM i.v. at the dose of 65 mg/mq over 1 hour on days 1, 8, and 15 followed after a 3-week interval by maintenance phase with bevacizumab at 10 mg/kg i.v. plus FTM 75 mg/mq every three weeks. Ten minutes before each infusion of FTM, a 5-HT3 receptor antagonist was administered for antiemetic prophylaxis. Antiepileptic drugs were given during the study period as medically indicated. Glucocorticoids were used to the dose necessary for neurologic stability. Study treatment was continued until disease progression, withdrawal of the patient, or unacceptable toxicity.

### 2.2. Study Assessment

MRI of the brain was uniformly adopted for tumor assessment and evaluation of the response. Magnetic resonance imaging was performed on a 1.5-T system (Optima MR450w, GE Healthcare, Milwaukee, WI) with dedicated 16-channel receive-only RF coils; slice thickness 4 mm and matrix size of 512 × 512 were used. Spin-echo (SE) sequences T1, T2, and FLAIR before contrast medium infusion in coronal plane (SE T2) and axial planes (SE T1, T2, and FLAIR) were performed; a three-dimensional volumetric T1-weighted sequence (fast-spoiled- gradient-echo sequence with fat saturation pulse), after the administration of gadopentetate dimeglumine contrast agent (Magnevist, Bayer Schering Pharma AG, Berlin, Germany), at a dosage of 0.1 mmol/kg of body weight, was performed. Both FLAIR and contrast-enhanced T1-weighted sequences were considered for the response assessment to treatment according to RANO criteria [17]. MRI examination was performed within 2 weeks before study begins. Further evaluations were performed after the completion of the induction phase every nine weeks during the maintenance phase or whenever disease progression was clinically suspected.

Toxicity was assessed before each drug administration by medical history, physical examination, hematology, and biochemistry. Adverse events were graded 1 to 4 according to the National Cancer Institute (NCI) common toxicity criteria (NCICT-CAE) version 3.0 [18]. Reduction in the bevacizumab dose was not permitted; dose delays were allowed for reversible and preventable toxicities.

FTM administration was omitted in case of grade 3-4 thrombocytopenia and/or neutropenia and grade 3-4 nonhematological toxicity except for nausea/vomiting. At recovery, treatment was resumed with a 25% dose reduction. Granulocyte colony-stimulating factor (G-CSF) was allowed. Data were collected for up to 12 months after the last bevacizumab/FTM infusion for the following specific adverse events: hypertension, proteinuria, arterial and venous thromboembolic events, congestive heart failure, central nervous system bleeding, other hemorrhages, wound-healing complications, and gastrointestinal perforations and fistulae.

### 2.3. MGMT Gene Promoter Methylation Analysis

Genomic DNA was isolated from one paraffin section of tissue collected at the time of initial diagnosis (Ex-Wax DNA Extraction Kit S4530, Chemicon), proteinase digestion lasting a maximum of 6 hours. DNA was denatured with sodium hydroxide and subjected to bisulfite treatment in a volume of 350 *μ*L (4.4 M sodium bisulfite and 20 mM hydroquinone) for five hours at 55°C and then purified. Unmethylated cytosine, but not its methylated counterpart, is modified into uracil by the treatment. The methylation-specific PCR was performed in a two-step approach. The results were confirmed in an independent experiment, starting with reisolation of DNA from the tumor.

### 2.4. Statistical Analysis

The primary endpoint of the study was the response rate. The study was designed according to A'Hern [18] to refuse a response rate of 10% when the true response rate was 30%. At a significance level of 5% with a power of 80% we needed 25 patients. The study would have been considered positive if at least 6 responses had been observed. Rates statistics were used to summarize pertinent study information. Rates are reported with their 95% confidence interval. The time to event analysis is measured from the start of treatment and is performed according to the Kaplan-Meier method. The disease control rate (DCR) was the sum of partial responses plus stable disease and clinical benefit (CB) was evaluated measuring steroid reduction and clinical status associated with the stability or response lasting ≥3 months. Progression free survival (PFS) was the time elapsing from the start of study treatment to the date of disease progression or death of the patient in the absence of documented disease progression. Overall survival (OS) was estimated from the first day of treatment to the date of death of the patient due to any cause. If a patient progressed/died, the progression and survival were censored at the time of the last visit.

## 3. Results 

### 3.1. Patients' Characteristics

Patients' characteristics are reported in Table 1. Twenty-six patients were recruited. Median age was 38 years (range 25–68) and median KPS was 80 (range 70–100). GBM was the most represented histotype (13 patients, 50% of cases); 15 (57.5%) patients performed primary surgery and all the GBM patients received concomitant radiotherapy and temozolomide followed by temozolomide (Stupp regimen) after diagnosis. Non-GBM patients received radiotherapy and temozolomide after diagnosis or progression of disease. Eleven patients (42%) had been treated with two previous chemotherapy lines (7 patients had continuative low dose temozolomide after first line temozolomide failure and 4 patients were given PCV polychemotherapy as second line). Patients received a median of 6 (range 2–15) cycles of bevacizumab plus FTM. MGMT status was assessable in 19 patients (73%), 14 on primary tumor tissue and 5 in the recurrent tumor tissue; MGMT resulted methylated in 10 (53%) cases and unmethylated in 9 (35%) cases.

### 3.2. Activity

**Figure 1:**
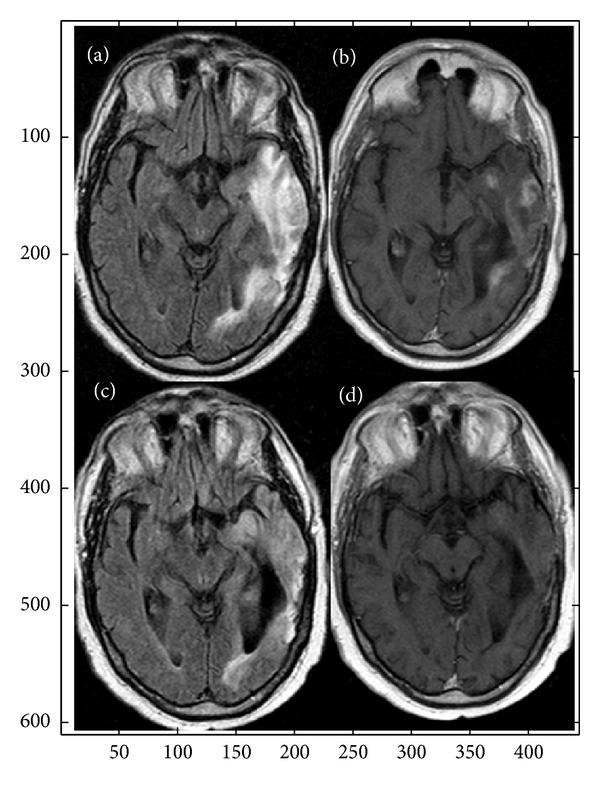
Before treatment MRI SE FLAIR (a) and T1 after contrast medium infusion (b) shows a large lesion in the left temporal lobe, hyperintense on FLAIR image with multiple focal enhancement areas after c.m. infusion. There is a compression on ventricle trigone. MRI FLAIR (c) and T1 (d) after contrast medium (c.m.), after treatment, show marked reduction of the hyperintensity area on the FLAIR sequence with disappearance of the enhancement areas on T1 sequence after c.m. The ventricle trigone is enlarged.

All patients were evaluable for activity. No case of complete response was observed; eight patients (31%) achieved partial response while 16 (61.5%) patients had disease stabilization, for a disease control rate of 92.5%. Two (7.5%) patients progressed, both at the end of induction phase. Responses or stability was observed in all histotypes (10 GBM, 8 AA, and 6 AOD/AOA) (Figure 1). In particular, partial responses were achieved in 2 GBM and 6 anaplastic gliomas, while stability was showed in 5 GBM and in 11 anaplastic gliomas.

Sixteen patients (60%) achieved clinical benefit; in all of them, a significant decrease of corticosteroid dose at the time of response was observed (median dose of dexamethasone from 12 to 4 mg every day; *P* = 0.001). Patients had no significant change of Mini Mental Status from the start to the end of treatment, while a significant benefit in terms of improvement of KPS was observed from the first to the last cycle of treatment (*P* = 0.02).

### 3.3. Progression Free Survival and Overall Survival

**Figure 2:**
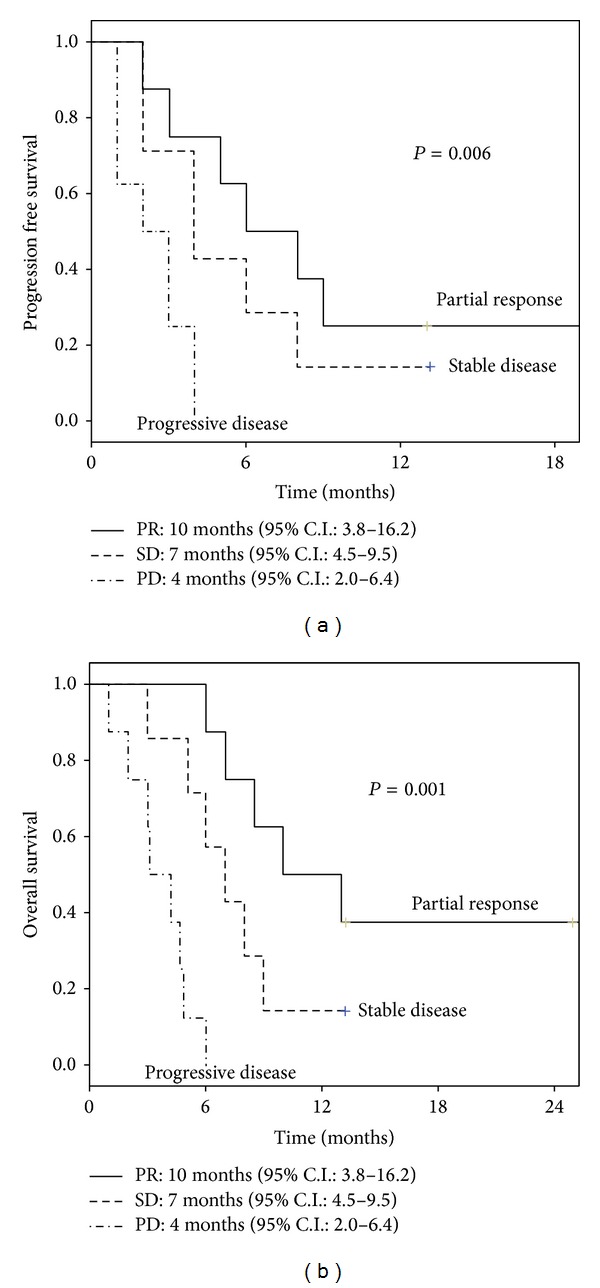
(a) Progression free survival according to response. (b) Overall survival according to response.

At a median follow-up of 6 months (range 2–13), the median PFS was 4 months (95% C.I.: 2.8–5.1). The rate of patients who were free of progression at 6 and 12 months was 23.1% and 11.5%, respectively. PFS differed with regard to response: 6 months (95% C.I.: 2.4–9.6) for responsive patients, 4 months (95% C.I.: 1.4–6.6) for patients achieving stable response, and 1 month (95% C.I.: ne) for progressive patients (*P* = 0.002) (Figure 2(a)). The median OS was 6 months (95% C.I.: 4.2–7.8). At 6 and 12 months, 49.2% and 20.5% of patients were alive, respectively. OS differed with regard to response: 8 months (95% C.I.: 5.1–10.9) for responsive patients, 6 months (95% C.I.: 4.7–7.3) for patients achieving stable disease, and 3 months (95% C.I.: 1.4–4.5) for progressive patients (*P* = 0.001) (Figure 2(b)). Regarding the different outcome related to histotypes, progression free survival in GBM and anaplastic gliomas was 3 months (95% C.I.: 0.7–5.4) and 4 months (95% C.I.: 3.2–4.8), respectively.

### 3.4. Activity according to MGMT

Among patients with assessment of MGMT methylation, 33% and 10% of responses were observed in MGMT methylated and unmethylated tumors, respectively.

### 3.5. Safety

All patients were evaluable for toxicity. Treatment-related adverse events are summarized in Table 2. The most common toxicities (all grades) were neutropenia in 6 (23%) patients, thrombocytopenia in 4 (15%) patients, and increase of AST and ALT in 3 (11.5%) patients. Grade 4 adverse events were neutropenia in 2 (8%) patients, leucopenia in 2 (8%) patients, and grade 3 thrombocytopenia in 2 (8%) patients. No case of severe anemia was observed. Hematological toxicity was mostly confined to the induction phase (data not shown). In fact in the maintenance phase only one patient (4%) developed a case of severe toxicity, namely, grade 3 thrombocytopenia. Severe nonhematological toxicity was uncommon: one case (4%) of grade 3 hypertransaminasemia and one case (4%) of nausea/vomiting. Toxicities associated with bevacizumab included grade 3 venous thromboembolic event occurring in 2 (8%) patients, grade 2 nose bleeding in 1 case (4%), grade 2 hypertension in 2 cases (8%), and grade 2 gastrointestinal (GI) perforation in 1 patient (4%), solved with medical treatment. Neither intracranial hemorrhage nor proteinuria occurred. The FTM dose was reduced by 25% in 4 patients. Causes for dose reduction were thrombocytopenia, neutropenia, and hypertransaminasemia. No drug-related deaths were reported (as being related to the study drug), nor any patients were permanently discontinued from the study due to toxicity. The reason for not proceeding into maintenance phase in six patients was disease progression.

## 4. Discussion

This is the final analysis of the observational, prospective study of bevacizumab and FTM combination in rMGs. Our finding showed that the association of bevacizumab plus FTM achieved a partial response of 31% with a disease control rate of 92% and a clinical benefit of 60%, reaching our preplanned goal; a 6-month PFS rate of 23% was observed.

Recently, several prospective and retrospective studies provided clinical data on bevacizumab activity both as single agent and in combination therapy, establishing this antiangiogenetic agent as a valuable and active treatment option in rMGs.

In phase II trials regarding the association of bevacizumab with chemotherapeutic agents including also irinotecan, the response rates ranged from 38% to 57% [12, 16] and activity achieved a percentage of about 32% [9]. Among the experiences with bevacizumab combination regimens in GBM, the 6-month PFS ranged from 37% to 50% [9, 12, 16, 19, 20]. Bevacizumab is also active as a single agent in patients with recurrent GBM reporting objective response rate ranging from 25% to 42% and 6-month PFS of 29–42% [12, 13, 21, 22]. Published data are heterogeneous in terms of efficacy in the different histotypes. In GBM tumors, bevacizumab obtained responses between 25% and 48%, while higher activity was seen in recurrent MGs (34%–68% of responses) [23].

A standard regimen has not been established for the recurrent/progressive MGs treatment. We chose fotemustine as nitrosourea to be combined with bevacizumab taking into account the rationale that bevacizumab could improve the activity of cytotoxic molecules without worsening primary toxicities of each agent. Several different experiences have been reported with this drug. Phase II trials demonstrated activity of nitrosoureas, such as FTM, in relapsing GBM patients previously treated with TMZ plus radiotherapy as first line treatment [4]. Recently Brandes et al. experienced use of FTM, in progressive GBM patients, at a conventional dose of 100 mg/mq for both induction and maintenance phase, reporting a 6-month PFS rate of 21%, a median OS of 6 months, and a partial response of 7% [5]. At the dose of 100 mg/mq, FTM has been reported to show a 30% of responses, a DCR of 62%, and a 6-PFS rate of 48–52% [24, 25]. The most frequent toxicities of full dose FTM were grade 3-4 thrombocytopenia and neutropenia observed in about 20% of patients. At lower dose, as demonstrated by Italian experiences in second-line treatment of rMGs, FTM did not reduce therapeutic activity; conversely, safety profile, mainly emathological, was improved [7]. All these results have defined FTM as an optimal therapeutic option in rMGs.

The only previous experience on the combination of FTM and bevacizumab in recurrent GBM was recently reported by Soffietti et al. Fotemustine was administered at a dose of 75 mg/m^2^ on days 1 and 8; bevacizumab was given at a dose of 10 mg/kg every two weeks; both were followed, after an interval of 3 weeks, by a maintenance phase of the combination with the same doses every 3 weeks. The authors showed a response rate of 52%, a median PFS of 5.2 months, and PFS rate at 6 months of 42.6%. The authors reported a median OS of 9.1 months and at 6 and 12 months the OS rates were 75.9% and 29.7%, respectively [26]. Although having the same schedule and dose, the different results of our study from those of Soffietti's trial might be due, probably, to the mixed histotype included in our population and to the patients' characteristics. Taking into account the previous chemotherapeutic lines administered, 42% of the patients were at the second recurrence and were heavily pretreated with temozolomide and combination regimens such as procarbazine, lomustine, and vincristine.

The comparison of our results with previous published data on the use of FTM as a single agent seems to show no further advantage in terms of both response rate and PFS when the drug is combined with bevacizumab.

Interestingly, in the present study bevacizumab plus FTM produced better results in terms of DCR (65.1%) than those reported with FTM as a single agent (47.5%), suggesting that the antiangiogenetic role of the drug, delaying the intravascular invasion and inhibiting the neovascularization [6], can result in a better disease control. Furthermore, our results confirm the relationship between response and patient outcome in terms of PFS and OS [7]. Overall these data highlight the importance of treating, if amenable, the MGs patients beyond the first relapse with further active drugs.

Within the second-line treatment of rMGs the safety profile of the regimen used is paramount. The combination of bevacizumab plus FTM used herein is well tolerated. Most frequent grade 3-4 toxicities were primarily related to chemotherapy. Bevacizumab-related toxicities were consistent with those reported in other trials. In our cohort 8% of patients experienced grade 3 venous thromboembolic events, while epistaxis, hypertension, and GI perforation were reported in a low percentage of patients (4%). In this experience no intracranial hemorrhage was seen, although heavy pretreated patients (43.5%) were included in the population.

On the other hand, in this analysis a significant improvement in terms of clinical benefit was reported. This translates in the achievement of a better clinical status, even in the absence of evident radiological responses. The clinical benefit plays an important role in MGs patients and could represent one of the best evaluation parameters of antiangiogenic drugs activity.

Preliminary results of the Dutch phase II (BELOB trial), randomized trial on firstly recurrent GBMs comparing bevacizumab plus lomustine versus either lomustine or bevacizumab alone, showed a better outcome in terms of 6-month PFS and OS for the combination. Primary endpoint for the combination treatment with bevacizumab and lomustine met the prespecified criterion for further investigation: 9-month OS rate of 59%, 6-month PFS rate of 41%, and median PFS of 4 months (at lower dose of lomustine) were achieved [27].

Further analysis is needed to clarify the real benefit of bevacizumab either as combination or as a single agent treatment. Many of the unanswered questions regarding the use of antiangiogenetic drugs in MGs are being addressed by ongoing clinical trials, namely, the MD Anderson randomized phase II trial [28], the recently opened EORTC 26101 study [29], and the ML25739 Italian study [30]; all of them are comparing the role of bevacizumab alone or in combination with nitrosourea with the attempt to define prospectively the benefit of the biological drug in comparison to chemotherapy.

## Figures and Tables

**Table 1:** Patient characteristics.

Characteristics	All patients (26)
Median age, years (range)	38 (range: 25–68)
Gender (%)	
Female	11 (42)
Male	15 (58)
Median baseline KPS	80 (70–100)
Histotype (%)	
GBM	13 (50)
Anaplastic astrocytoma	7 (27)
Anaplastic oligodendroglioma	2 (8)
Anaplastic oligoastrocytoma	4 (15)
Prior surgery (%)	
Biopsy	11 (42.5)
Partial resection	11 (42.5)
Total resection	4 (15)
Prior radiotherapy	26 (100)
Second surgery	7 (27)
Prior lines of chemotherapy (%)	
1	26 (100)
2	11 (42)
Type of prior chemotherapy (%)	
TMZ	26 (100)
PCV	4 (15)
MGMT gene promoter methylation status (%)	
Evaluable	19 (73)
Unmethylation	10 (53)
Methylation	9 (47)

KPS: Karnofsky performance status; TMZ: temozolomide; PCV: procarbazine, carmustine, and vincristine; MGMT: methylguanine methyltransferase.

**Table 2:** Grade 3-4 toxicities per patient (total: 26).

	Number of patients (%)
Grade 3-4 haematologic toxicity	
Neutropenia	2 (8)
Leucopenia	2 (8)
Thrombocytopenia	2 (8)

Grade 3-4 nonhaematologic toxicity	
Venous thromboembolism	2 (8)
CNS hemorrhage*	1 (4)
Hepatic^∧^	1 (4)
Emesis	1 (4)

*Asymptomatic central nervous system hemorrhage.

^∧^Transaminase increasing.
